# Colloidal nitrogen is an important and highly-mobile form of nitrogen discharging into the Great Barrier Reef lagoon

**DOI:** 10.1038/s41598-018-31115-z

**Published:** 2018-08-27

**Authors:** Jonathan D. Judy, Jason K. Kirby, Mark Farrell, Mike J. McLaughlin, Scott N. Wilkinson, Rebecca Bartley, Paul M. Bertsch

**Affiliations:** 1 Land and Water, Commonwealth Scientific and Industrial Research Organisation (CSIRO), Waite Campus, Waite Road, Urrbrae, 5064 South Australia Australia; 2 Soil and Water Sciences Department, University of Florida, 1692 McCarty Drive, Gainesville, 32603 Florida USA; 3 Agriculture and Food, The Commonwealth Scientific and Industrial Research Organisation (CSIRO), Waite Campus, Waite Road, Urrbrae, 5064 South Australia Australia; 4 School of Agriculture Food and Wine, The University of Adelaide, Waite Campus, Waite Road, Urrbrae, 5064 South Australia Australia; 5 Land and Water, Commonwealth Scientific and Industrial Research Organisation (CSIRO), GPO Box 1700, Canberra, 2601 Australian Capital Territory Australia; 6 Land and Water, Commonwealth Scientific and Industrial Research Organisation (CSIRO), 41 Boggo Road, Ecosciences Precinct, Dutton Park, 4102 Queensland Australia

## Abstract

Soil-borne colloids have been linked to long-distance transport of radionuclides, metal(loid)s and nutrients. Colloid-associated nitrogen (N) will have different mechanisms of biogeochemical cycling and potential for water-borne transport over longer distances compared to dissolved N. The role that colloids play in the supply and mobility of N within catchments discharging into the Great Barrier Reef (GBR) lagoon is unexplored. Here, we examine water-dispersible clay (WDC) from soil samples collected from gullies and agricultural drains within three different land uses (sugarcane, non-agricultural land and grazing) within the Townsville area. The proportion of soil N associated with WDC was inversely correlated with total soil N, with up to 45% of the total soil N being colloid-associated in low N gully soils. Within the <0.45 µm fraction of the WDC, only 17–25% of the N was truly dissolved (<3 kDa) at the gully sites compared to 58% in the sugarcane sites. Our results demonstrate the importance of colloidal N and the inaccuracy of assuming N < 0.45 µm is dissolved in the sampled areas, as well as providing an alternate explanation for the large amounts of what has previously been defined as dissolved inorganic N in runoff from non-fertilized grazing land. In particular, they describe why non-fertilized land uses can contribute significant N < 0.45 µm, and why catchment models of nutrient export based on soil N concentrations can over-estimate loads of particulate nitrogen derived from monitoring data (N > 0.45 µm). The findings suggest that managing soil erosion may also contribute to managing N < 0.45 µm.

## Introduction

Since European settlement of Australia in the late 18^th^ century^1^, sediment and nutrient loads to the Great Barrier Reef (GBR) marine zone have increased dramatically as a result of land-use changes such as urbanization, mining and agricultural intensification^1–3^. During this time period, total suspended solids (TSS), total nitrogen (N) and total phosphorus (P) delivery to the GBR lagoon has increased 5.5, 5.7, and 8.9 fold, respectively^3^. These losses are particularly large during flood events, with nutrient and sediment discharges increasing up to 100 fold compared to non-flood conditions^4^. This flux has been linked to eutrophication, loss of marine biodiversity, crown-of-thorns starfish outbreaks, reduced fishery productivity, and a dramatic reduction in GBR coral cover^3,5,6^.

In studies by Wilkinson *et al*. (2013), fallout radionuclide-facilitated sediment tracing suggested that up to 77–95% of sediment <10 µm in diameter exported from the Burdekin River Basin (draining 129,700 km^2^ of North Queensland) was derived largely from sub-surface gully and streambank erosion^7,8^. To what degree this exported sediment is comprised of colloids is currently a key knowledge gap. Colloids, defined by the International Union of Pure and Applied Chemistry (IUPAC) as stable suspensions of well-dispersed particles <1 µm, have been linked to long-distance transport of metal(loid)s, radionuclides, nutrients and organic contaminants in aquatic and terrestrial environments^9–12^. Stable colloidal suspensions are generated in the soil through precipitation of secondary phases, release of colloidal particles as a result of dissolution of cementing agents, physical disruption resulting from rainfall or other water flows^12^ and electrostatic enhancement of particle stability *via* changes in soil chemistry^12^. Stable colloids are subsequently available to participate in the vertical transport of inorganic and organic material through the soil profile or delivered to surface waters via erosion and runoff^12–14^.

Upon migration within catchments, colloids can be highly stable and transported long distances^9,12^. For example, Lewis *et al*. (2013) examined the trapping efficiency of the Burdekin Falls Dam as a function of particle size, determining that no particles <0.5 µm and only 50% of those between 0.5 and 5 µm were trapped by the dam^15^. Bainbridge *et al*. (2012) examined the export of sediment and nutrients from a near-coastal location on the Burdekin River out into the GBR lagoon as a result of a flood plume^16^. Fine silt (3.9–15.6 µm) and clay (<3.9 µm) particles were transported up to 100 km north of the Burdekin River mouth. As a result of this long-distance mobility, colloids have the potential to be a vector by which nutrients are transported longer distances than would otherwise be possible^17^, entering the GBR lagoon and possibly contributing to degradation of water quality and ecosystem health^5,6,18^.

Research on N transport in the GBR catchment area commonly examines water samples separated into <0.45 µm and >0.45 µm fractions to operationally determine “dissolved” and particulate fractions (total N minus 0.45 µm filtered). This approach is largely a product of operational analytical concerns, as investigating the <0.45 µm fraction in detail is currently impractical on a routine basis. For routine sampling, investigators rely on sampling techniques that can be readily deployed for numerous samples and in the field. These techniques include classic separation technologies such as 0.2 and 0.45 µm syringe filters and metal screens, among others. Investigating what proportion of dissolved inorganic N (DIN) and dissolved organic N (DON) reported in past GBR catchment area studies was actually colloidal or pseudo-colloidal (i.e. sorbed to colloids) has not been attempted on even a limited basis.

Modelling of dissolved nutrient fluxes has been based on monitoring data, and these efforts have identified grazing lands and sugarcane cultivation as the primary sources of DIN flux to the GBR, with sugarcane cultivation contributing 3,857 tons yr^−1^ (37% of total DIN load) and grazing contributing 2,952 tons yr^−1^ (28% of total DIN load)^19,20^. In the Burdekin region, the most recent available reports estimate that grazing and sugarcane cultivation contribute 36 and 44% of the total DIN load, respectively. Dissolved N (DN) from these different land uses has different characteristics, with DN from grazing lands existing predominately as DON (38% of total nitrogen (TN) load to GBR lagoon^19^) and DN from sugarcane cultivation existing predominately as DIN (25% of TN load to GBR lagoon^19^)^21,22^. Whether more DIN is exported from grazed savannah than is exported from ungrazed savannah is still being investigated. Earlier work that did consider ungrazed land estimated the concentration of DIN in runoff from grazed land to be twice that from runoff from ungrazed land^23^.

Modelling of particulate nutrient exports from catchments has been based on measured soil nutrient concentrations, but has repeatedly tended to over-estimate particulate nutrient exports relative to river load monitoring data^3,23^. While this over-estimation may be attributable to incorrect estimates of the nutrient concentrations of soil actually delivered to streams, the contribution of soil-borne colloids (colloids generated from soil components) has not been considered in load modelling and monitoring to date and may provide an alternate explanation for the discrepancies between modelling and monitoring data.

Here we investigate if soil-borne colloids are an important component of N in selected sample sites within the Burdekin River Basin area. The Burdekin River Basin, first settled by Europeans in 1862^1^, drains 129,700 km^2^ of North Queensland, affecting approximately 246^24^ coral reefs and represents 34% of the total GBR catchment, the management of which is crucial for the future health of the GBR^20^. Land use in the Burdekin consists of 2% sugarcane cultivation and 91% cattle grazing, both of which are major revenue sources for the region^20^. The Burdekin has been previously characterized as posing a high risk to GBR water quality and ecosystem health as a result of agricultural runoff which includes herbicides, DIN and fine sediment^20^.

In this study, we characterize the elemental composition, mineralogy, and N content of the water-dispersible clay (WDC) from soil samples collected from sugarcane, grazing and non-agricultural areas, as well as the <0.45 um and <3 kilodalton (kDa) fractions within the WDC. The objective of this work is to clarify the degree to which N from different land uses is colloidal or colloid-associated. As it is currently unclear to what degree colloids are responsible for long-distance transport of nutrients from different land uses within the GBR catchment area, the results of this research will help refine our understanding of N fluxes into the GBR lagoon and will contribute to the development of improved models and management practices.

## Results

**Figure 1:**
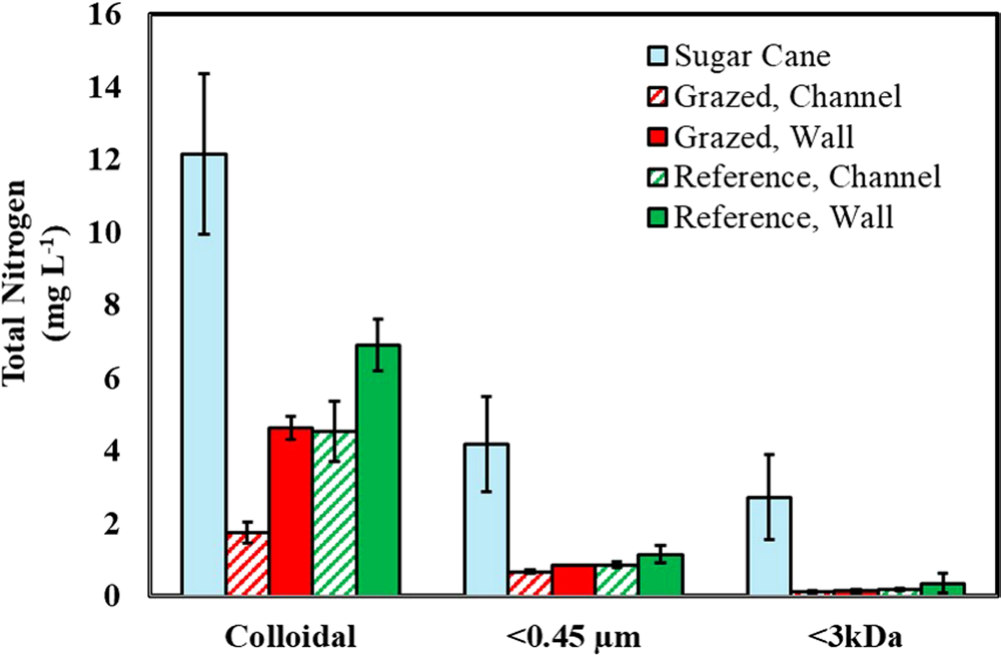
Nitrogen concentrations measured in water-dispersible clay (WDC) suspensions, as well as in <0.45 µm and <3 kDa N filtrates. Error bars represent standard error.

**Figure 2:**
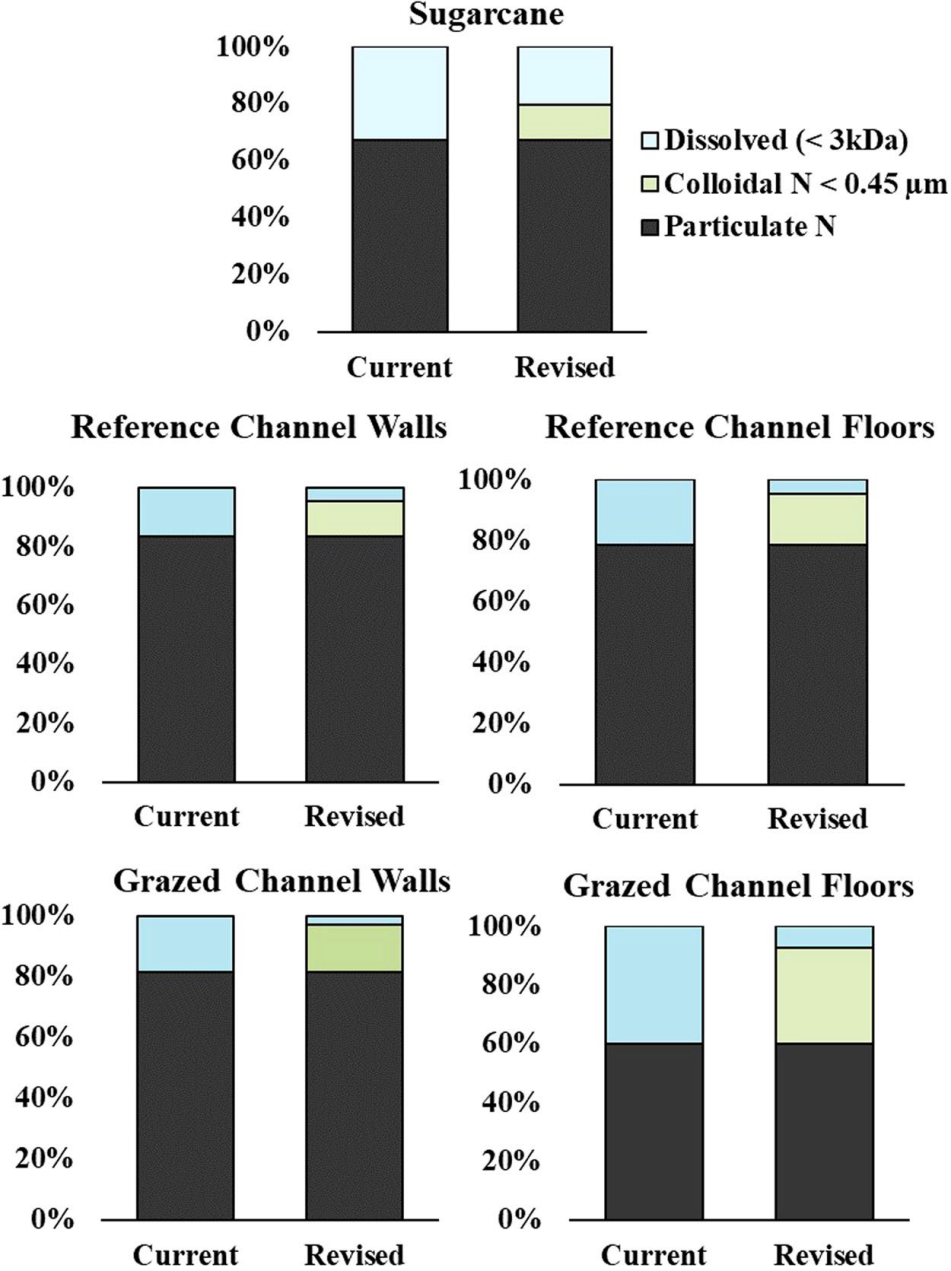
Mean percent N in dissolved (<3 kDa), colloidal N < 0.45 µm and particulate phases within water-dispersible clay (WDC) extracts for (top) sugarcane, (middle) reference gully (wall and channel floor) and (bottom) grazed gully samples (walls and channel floor). For each site type, the left bar (current) represents N fractionation assuming all N < 0.45 µm is dissolved and the right bar (revised) incorporates measurements of colloidal <0.45 µm and dissolved (<3 kDa) determined in this study.

The WDC, <0.45 µm fraction and the <3 kDa fraction from the samples from the sugarcane sites all contained significantly higher concentrations of N than the respective fractions in the gully sites (Pr > F = < 0.001 for WDC and <3 kDa fractions and Pr > Chi-Square = <0.05 for the <0.45 µm fraction; Fig. 1). Within the <0.45 µm fraction, 58% of the N was dissolved (<3 kDa) in the sugarcane WDC extracts and only 17–25% for the gully sites (Fig. S1). Comparing these results to those that would have been obtained assuming that all <0.45 µm N is dissolved, the degree to which this assumption overestimates the dissolved N pool, especially for the gully sites, is clearly demonstrated (Fig. 2).

**Figure 3:**
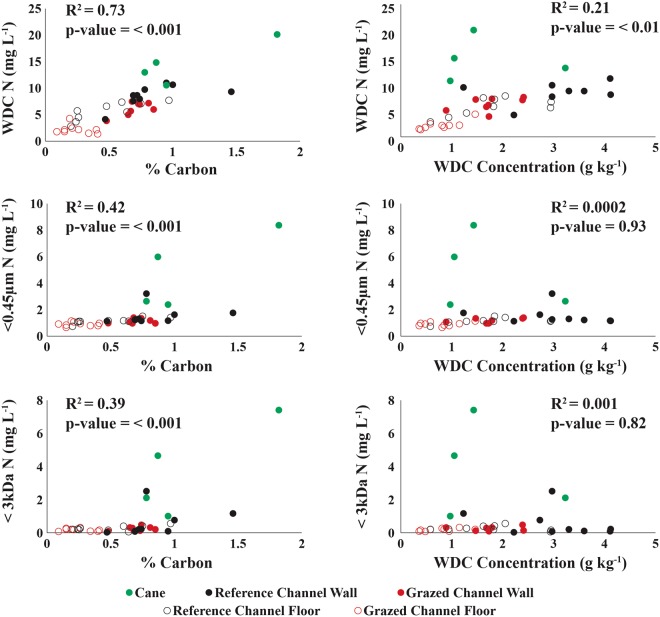
Analysis of the relationship between soil carbon (left) and water-dispersible clay (WDC) concentration (right) for WDC N (top), <0.45 µm N (middle) and <3 kDa N (bottom).

**Figure 4:**
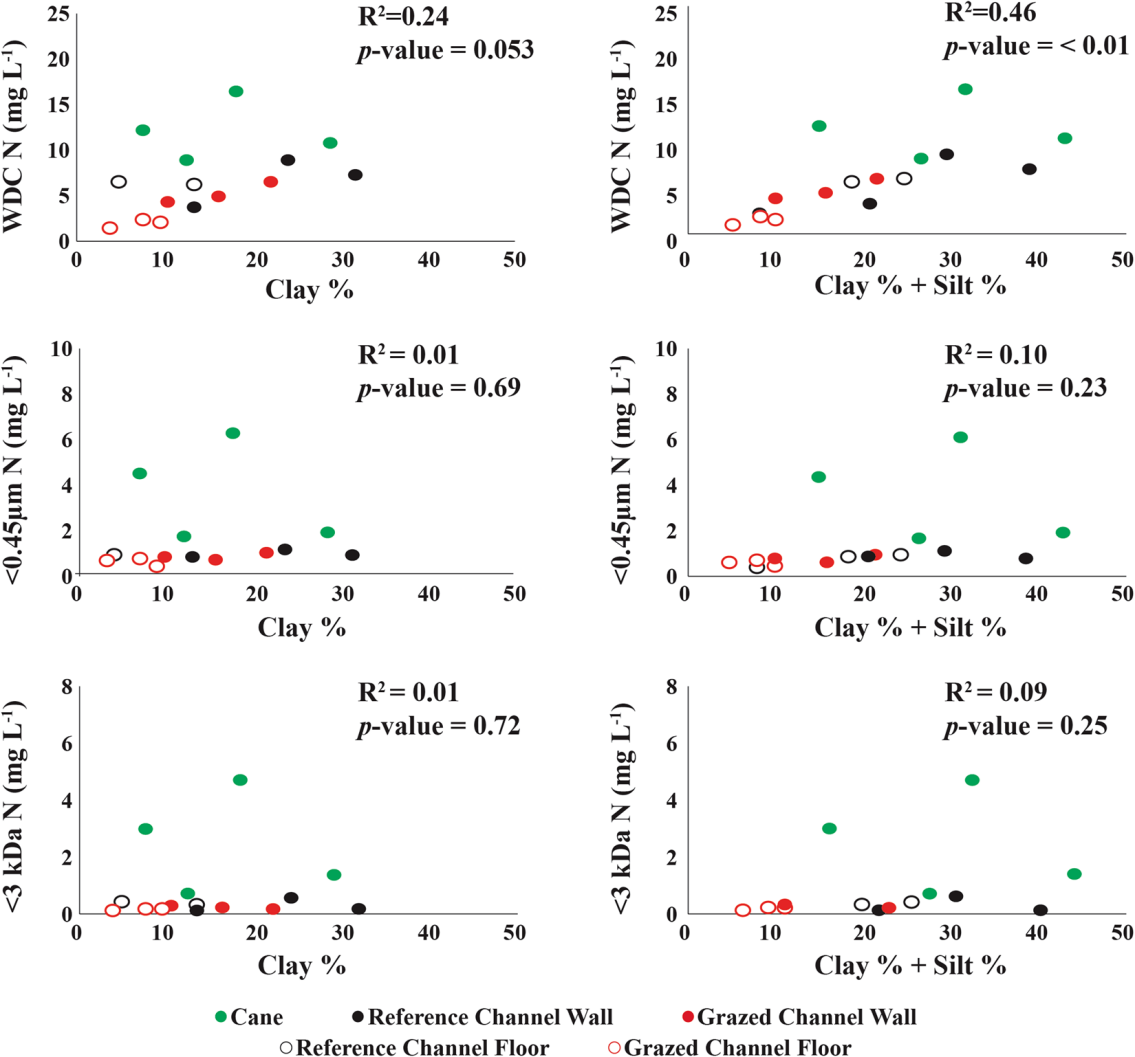
Analysis of the relationship between soil clay percentage (left) and soil clay percentage plus soil silt percentage (right) for water-dispersible clay (WDC) N (top), <0.45 µm N (middle) and <3 kDa N (bottom).

**Figure 5:**
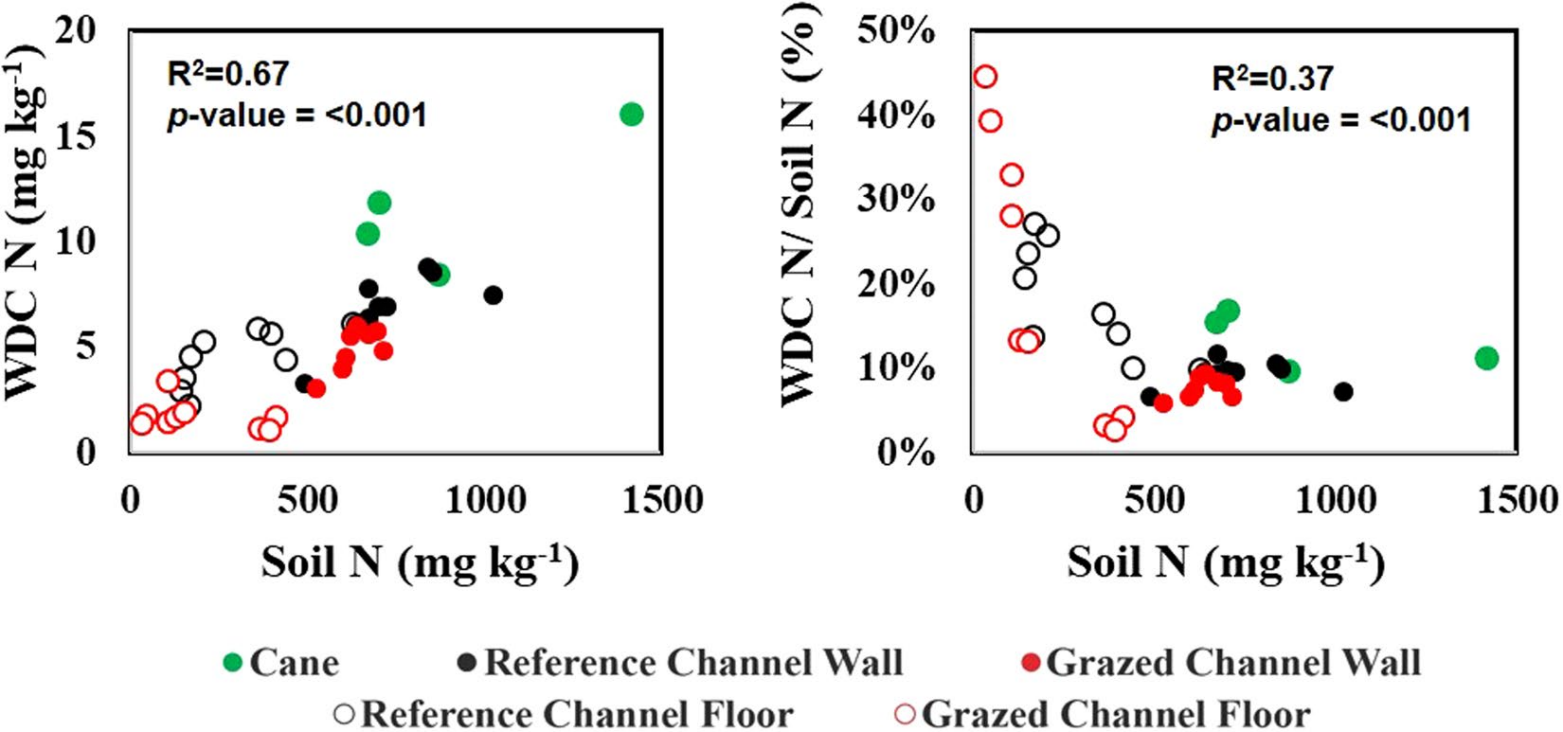
Analysis of the relationship between soil N and water-dispersible clay (WDC) N (left) as well as the percentage of soil N that is present in the WDC (right).

As reported in earlier work examining colloid-facilitated transport of contaminants^25–27^, the concentration of N in the WDC was strongly correlated with % C (Pr > F = < 0.001), WDC concentration (Pr > F = < 0.01), soil clay % plus silt % (Pr > F = < 0.01), and soil N content (Pr > F = < 0.001) across all sampling sites, with % C having the strongest correlation to WDC N (Figs 3 and 4). However, the relationship between WDC concentration, soil clay % plus silt % and WDC N is not significant in the <0.45 µm and <3 kDa size fractions, suggesting that particles >0.45 µm are responsible for a large component of colloidal transport. Furthermore, the proportion the N in soil that was associated with WDC was inversely correlated with total soil N (Pr > F = < 0.001; Fig. 5), with up to 45% of total soil N present in the samples being colloidal or colloid-associated for some of the lower N gully samples.

**Figure 6:**
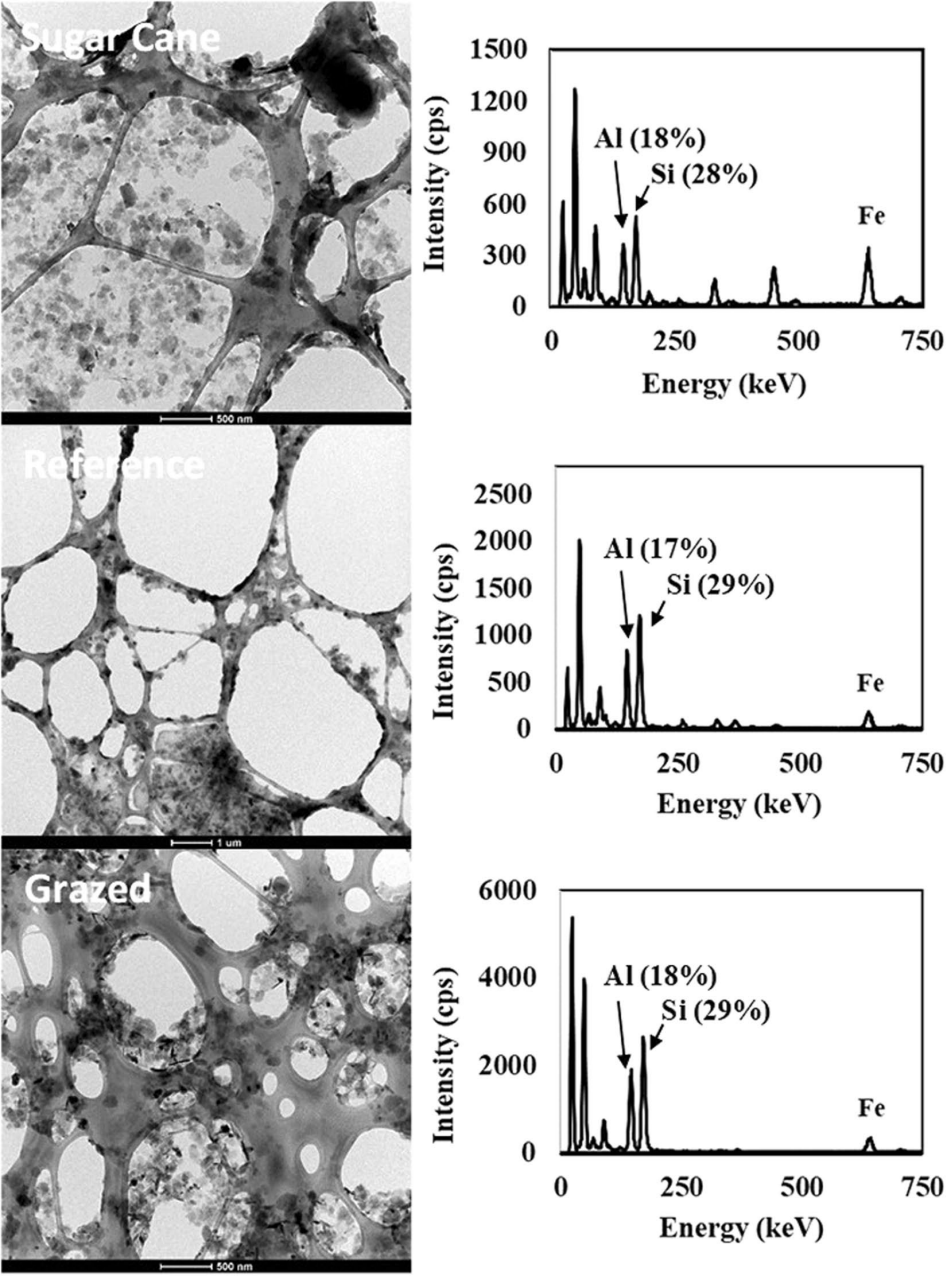
TEM micrographs (left) and EDS spectra (right) of particles present in <0.45 µm filtrates of water-dispersible clay (WDC) collected from sugar cane (top), reference (middle) and grazed (bottom) gully channel floor samples.

Characterization of the WDC from the different sites revealed that the WDC from the gully channel floors were distinct compared to the WDC from the cane sites and gully channel walls, having significantly lower N content (Table 1). There were no significant differences between the sugarcane WDC and the gully wall WDC except at the reference sites, which contained a higher WDC concentration and smaller average particle size. No statistically significant differences were measured in WDC electrophoretic mobilities (Pr > Chi-square = 0.38; Table 1). DLS measurements of particle hydrodynamic diameter of the WDC suspensions indicated that while the sugarcane WDC particles were larger than those present in the reference samples, they were not significantly different in size from the WDC in the grazing sites (Table 1). XRD analyses revealed that sugarcane sites had a larger proportion of dioctahedral smectite and a smaller proportion of kaolinite than were present in the gully WDC (Table S2). However, TEM analysis confirmed the presence of solids within the <0.45 µm fraction. The Si:Al ratios of the solids within this size fraction were similar between the different land uses (Fig. 6). The compositional similarity of the solids present in the <0.45 µm fractions suggests that certain minerals contained in nano-scale weathering products form stable suspensions and, therefore, are more concentrated in the 0.45 µm fraction, a finding consistent with previous research^12^.

**Table 1:** Analysis of selected sample characteristics as a function of sample site type.

Characteristic	Sugarcane	Reference		Grazed	
		Channel floor	Channel wall	Channel floor	Channel wall
	Mean ± sd	Mean ± sd	Mean ± sd	Mean ± sd	Mean ± sd
Carbon (%)	1.11 ± 0.48^a^	0.49 ± 0.25^bc^	0.83 ± 0.22^ab^	0.24 ± 0.12^c^	0.71 ± 0.08^abc^
WDC (g kg^−1^)	1.67 ± 1.06^b^	1.78 ± 0.74^ab^	3.03 ± 0.65^a^	0.78 ± 0.19^b^	1.79 ± 0.38^ab^
Electrophoretic mobility (µmcm Vs^−1^)	−1.88 ± 0.43	−1.64 ± 0.07	−1.57 ± 0.06	−1.51 ± 0.34	−1.49 ± 0.03
Z-average diameter (nm)	525.7 ± 27.0^a^	416.8 ± 25.1^b^	409.8 ± 33.4^b^	550.3 ± 71.9^a^	523.7 ± 38.6^a^
Nitrogen (mg kg^−1^)	913.7 ± 346.6^a^	294.0 ± 130.9^bc^	735.6 ± 109.6^a^	191.9 ± 170.3^c^	640.1 ± 39.8^ab^

For gully channel samples, the three cross-sections were averaged to get a single data point for each gully. Data expressed as mean ± one standard deviation (SD = standard deviation; *n* = 3 for gullies and 4 for sugar cane). Electrophoretic mobility and Z-average diameter were measured on water dispersible clay (WDC) suspensions whereas other data was collected on whole samples. There were no significant differences between treatments in electrophoretic mobility (Pr > F = 0.34). Means with the same superscript letter are not significant different at α = 0.05.

## Discussion

Although many studies have linked soil-borne colloids to the facilitated transport of radionuclides^9^, metals^17,28,29^, phosphorus^26,30–32^, and organic contaminants^10^, there has been limited work undertaken examining either colloid-facilitated or colloidal N transport. Within the body of literature on N transport that does exist, studies have reported that colloidal N is an important component of N flux^27^. One study examining N transport in the Southeastern United States reported that colloids were involved in the transport of N and that colloid-bound N could desorb from particulates upon entering an estuary^17^. Other work has reported that organic C preferentially partitions to colloids over larger particulates in aqueous suspensions and that this enrichment results in a high concentration of colloidal N and a colloidal C/N ratio more similar to microorganisms than soil^33^.

The work presented here demonstrates that colloid-associated N is an important component of total N present in the sites sampled, with nearly half of the soil N being colloid-associated at some of the low N sites (Fig. 5). Furthermore, this study demonstrates that colloidal N, colloid-associated N and/or high-molecular weight DON, which are routinely categorized as “dissolved”, comprise an important component of the N flux in the sites sampled. These findings provide an alternate explanation as to how non-fertilized grazing land can contribute large amounts N to aquatic environments, exports which have alternatively been attributed to high mineralization rates resulting from efficient bacteria and high soil temperatures^3,23^. Even in sampling sites (i.e. the sugarcane sites in this study) where the majority of the <0.45 µm N in the WDC suspensions is dissolved, a major component is colloidal or pseudo-colloidal. However, further work on the potential reaction, transformation and delivery of these colloids in both freshwater and marine environments is required. Collectively, these findings may contribute to improvements in the accuracy of water quality data for end of river systems delivered to marine biogeochemical models such as eReefs (https://ereefs.org.au/ereefs). The findings also have implications for the setting and evaluation of water quality targets^34^, as the attribution of DIN (<0.45 µm) to various land uses and soil types may need to be re-evaluated. This work also provides an important insight into the current research that is evaluating the bioavailability of various forms on N in the GBR catchments.

The degree to which colloidal or colloid-facilitated transport of N into the GBR lagoon could be feasibly reduced is unclear, beyond reducing soil erosion. Colloidal/colloid-facilitated transport of contaminants in porous media is reduced as media particle size becomes smaller. This is the result of colloid straining, a process that occurs when media pore size is so small that it restricts colloidal passage^35^. Researchers have explored exploiting this process to reduce colloid-facilitated transport of radionuclides through the introduction of compacted bentonite clay, effectively filtering the colloids^36^. An alternative and, in this specific example, likely more practical potential management strategy would be to introduce material that would destabilize/aggregate colloids. Colloids are strongly destabilized by the presence of multivalent ions^37^, and the addition of material such as gypsum (CaSO_4_ * 2H_2_O) to erosional features such as gullies and cane drains may result in reduced colloidal flux, though this was not assessed in this study.

Whether the colloid-associated N is present as an insoluble organic or inorganic phase, a large molecular weight (>3 kDa) dissolved phase, or is N in an organic or inorganic form that is strongly adsorbed to colloidal particles was not determined here and is a critical area for future study^38^. Another area for future study would be examining N fractionation in surface waters discharging into the GBR lagoon and within the GBR lagoon itself, as this study does not examine the behavior of WDC associated N once it enters high-ionic strength waters. While we expect that a large proportion of the larger colloids will flocculate once they enter high ionic strength waters, we hypothesize that some fine colloids will remain suspended, a hypothesis supported by work reporting clay and silt-sized particles in waters 100 km north of the mouth of the Burdekin River^16^. Regardless, the results of this study suggest that soil-borne colloids may play an important role in the transport of N within the catchments that discharge into the GBR marine zone and future studies examining colloid-associated N in surface waters within the Burdekin basin as well as in the lagoon itself are necessary.

## Methods

### Sampling locations

**Figure 7:**
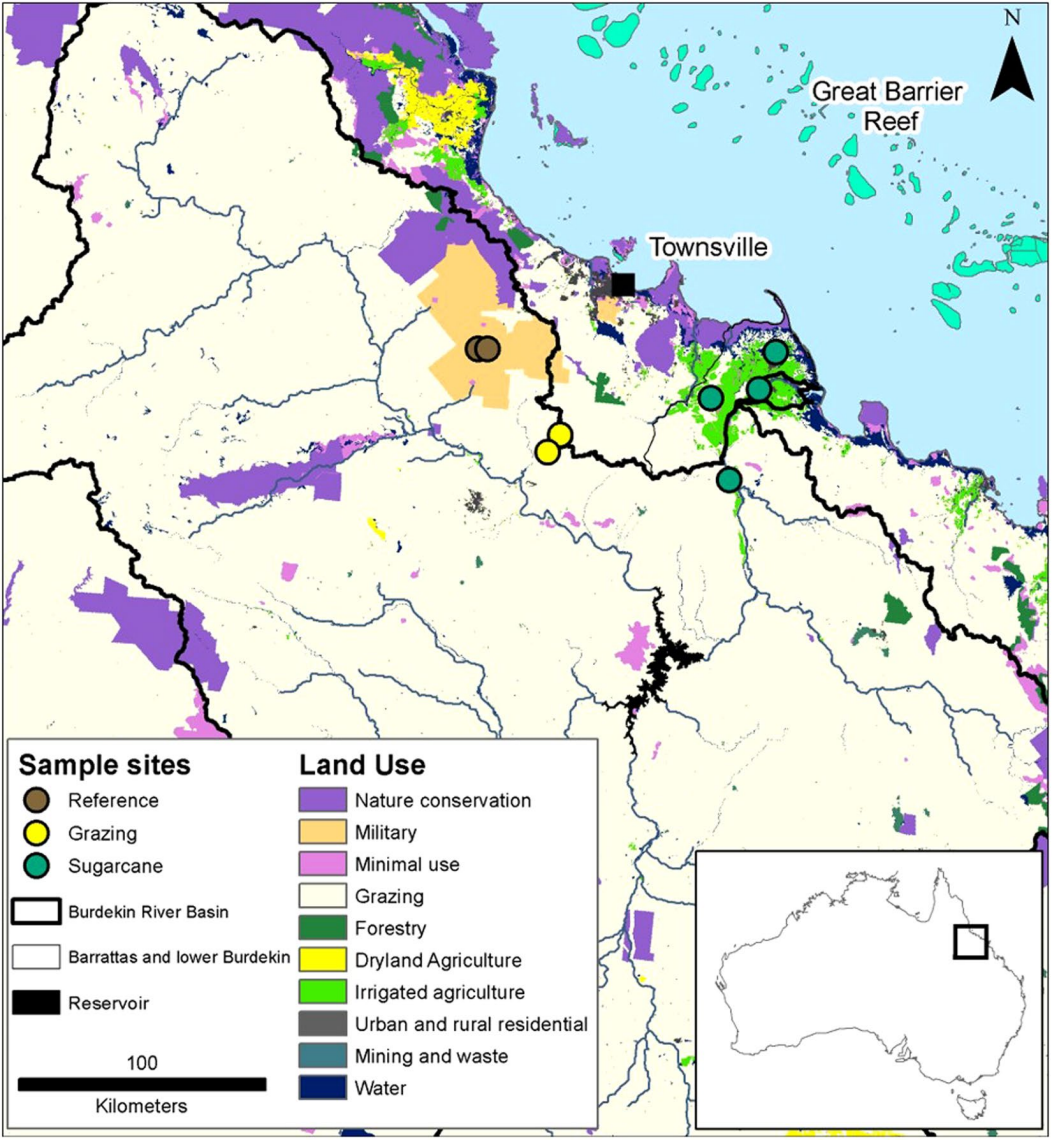
Map of Burdekin River Basin and sampling locations. Three grazed and reference sites were sampled (sites close to each other on scale of this map and markers for third site for both grazed and reference overlap), whereas four sugarcane sites were sampled. Map generated using ArcGIS 10.4 (www.arcgis.com).

All sampling sites were located near Townsville in Queensland, Australia (Fig. 7). Sample site characteristics including yearly rainfall, gradient, soil type, parent geology, predominant ground vegetation, and fertilizer application rates can be found in Table S1 in supporting information. Samples were collected from three gully sites within the Main Creek sub-catchment (11 km^2^ catchment area), two sites within the Weany Creek sub-catchment (13 km^2^), and one site within the Wheel Creek sub-catchment (11 km^2^) drainage basin. (Samples from sugarcane sites were collected from areas adjacent to cane fields from exposed soil (top 10 cm) being transported off the cane fields. Three sites were from roadside cane drains while the fourth was from exposed soil being transported from a cane field, but not within a purposefully built drain. Sampling sites were located within the Lower Burdekin (4,830 km^2^) and Barratta Creek (1,830 km^2^) catchments. Samples consisted of several sub-samples collected and bulked at each sampling location.

The gullies within the Main Creek site are located on the Department of Defense Townsville field training area which has not been used for commercial cropping or grazing for >15 years, although these areas had been used for grazing prior to that. These non-agricultural sites will hereafter be referred to as reference sites. Sampling sites within the Weany and Wheel Creek sub-catchments are located on land used for cattle grazing for ~100 years^39^. Three gully sites were sampled for each of grazing and reference land uses and 4 sampling locations for sugarcane. Each erosion gully was sampled at three cross-sections at different distances from the gully head that were averaged to determine values for each gully. Each sample consisted of a bulk sample of gully channel sediment composited from several sub-samples collected from the top 10 cm of the gully floor at the cross-section location. A bulk sample was also taken from the gully walls at each cross-section, again being comprised of several subsamples of gully wall material collected at the cross-section location. All samples were air-dried after collection and sieved to <2 mm. Samples were analyzed for total C and N by elemental analysis (Tru-Mac CNS analyzer, LECO Corporation, St. Joseph, Michigan, USA).

### Water dispersible clay collection

WDC was used as a proxy for soil-borne mobile colloids and was collected using a modified micropipette method as described by Kaplan *et al*.^11,12,40^. Four grams of the <2 mm sieved samples were weighed into 50 mL centrifuge tubes into which 40 mL of deionized water (DI) were added. Samples were shaken overnight. Samples were allowed to settle for 1 h and 50 min, after which a sample of ~2.5 mL was collected from the top 2.5 cm of the suspension.

### Water dispersible clay characterization

The amount of WDC within each sample was determined by drying samples of each suspension and weighing to the nearest 0.1 mg. The particle size distribution of the WDC suspensions was determined by dynamic light scattering (DLS) and the electrophoretic mobility determined by phase analysis light scattering (PALS) using a Nano-ZS Zetasizer (Malvern Instruments Ltd, Worcestershire, UK). The mineralogy of oven-dried samples of WDC was analyzed by X-ray diffractometry (XRD) on powder mounts using a X’Pert Pro Multi-purpose Diffractometer (PANalytical, Almelo, The Netherlands). This XRD uses a Fe filtered Co Kα radiation, auto divergence slit, 2° anti-scatter slit and fast X’Celerator Si strip detector. The diffraction patterns were recorded in steps of 0.016° 2θ with a 0.4 second counting time per step and logged to data files for analysis. Soil texture analysis was determined by dispersion, wet and dry sieving and by pipette sub-sampling. The sample was dispersed by probe-sonication, after which organic matter, carbonate and soluble salts were removed using hydrogen peroxide, and washes of sodium hexametaphosphate/sodium carbonate, respectively^41^. The sample was then transferred into a sedimentation cylinder and pipette subsamples collected at times and depths calculated to correspond to particle size class boundary equivalent spherical diameters.

The mass of WDC, particle size distribution, and electrophoretic mobility were analyzed for three technical replicates from each sampling location, including each cross-section from each gully. For gully channel samples, the three cross-sections were averaged to get a single data point for each gully. XRD and soil texture analysis was conducted on one sample from each site. For the gully sites, these analyses were conducted on the sample collected from the cross-section furthest from the gully head.

### Nitrogen fractionation

The proportion of N present in different size fractions within WDC samples was assessed by analyzing samples of WDC along with aliquots of WDC sample filtered using either a 0.45 µm cellulose acetate filter or a 3 kDa centrifuge filter (EMD Millipore, Darmstadt, Germany). The <0.45 µm size fraction would be expected to contain <0.45 µm solids, high molecular weight (>3 kDa, e.g. proteins and humic/fulvic acid polymers) dissolved molecules and low molecular weight dissolved molecules (<3 kDa) whereas the <3 kDa fraction would only contain low molecular weight dissolved molecules^42^. The WDC would contain the same N sources as the <0.45 µm size fraction as well as all N associated with colloids >0.45 µm. While we will use the above operational definitions of the N types and sizes contained within each size fraction, the particles within the filtered fractions are likely to be smaller than the filter pore sizes, as clogging of pores during filtration may reduce the effective pore size of the filters. Nitrate spike recovery was assessed for the 3 kDa filters resulting in a recovery of 92.4% ± 2.9% (mean and standard deviation; *n* = 4). WDC, <0.45 µm and <3 kDa samples were analyzed by catalyzed high-temperature combustion using a Thermalox total organic C (TOC)/total N (TN) analyzer (Analytical Sciences Ltd., Gloucestershire, UK). Three subsamples were analyzed and averaged for each fraction from each sampling location. As with the physical characterization, the three cross-sections were averaged to get a single data point for each gully. Each analytical run included filtration blanks, the mean N content of which was subtracted from sample data, and drift-detection samples.

### Transmission electron microscopy sample analyses

The morphology and elemental composition of solids present in the <0.45 µm fraction were analyzed via TEM to confirm that the N present in the <0.45 µm fraction that was larger than 3 kDa was at least partially associated with a solid phase, as opposed to being composed of high molecular weight dissolved material. WDC samples were filtered using 0.45 µm cellulose acetate filters and direct-mounted onto 300 mesh lacey C coated copper TEM grids for TEM/EDS analysis. These samples were analyzed using a Tecnai G2 Spirit (FEI, Hillsboro, Oregon, MA, USA) TEM or a Philips CM200 (FEI, Hillsboro, Oregon, MA, USA) using an Oxford X-Max SDD X-Ray detector.

### Statistics

All statistical analyses were performed using SAS 9.4. Comparisons between means were conducted using one-way analysis of variance (ANOVA) and least significant differences (LSD) pair-wise means comparisons (PROC ANOVA procedure in SAS). Normality and homoscedasticity were tested using Shapiro-Wilk’s and Bartlett’s tests, respectively. Non-normal and/or non-homogenously varied data were log transformed and re-tested. If data were still not normal and/or homogenously varied, data were then analyzed via a Kruskal-Wallis test and, if necessary, pairwise Mann-Whitney U-tests. Correlations between variables were analyzed using general linear regression analysis (PROC GLM procedure in SAS).

## Electronic supplementary material

Supporting information

## Data Availability

The datasets generated during and/or analyzed during the current study are available from the corresponding author on reasonable request.
