# Supplementary material for: Colloidal nitrogen is an important and highly-mobile form of nitrogen discharging into the Great Barrier Reef lagoon
Source: Sci Rep. 2018 Aug 27;8:12854. doi: 10.1038/s41598-018-31115-z (PMC6110740; doi:10.1038/s41598-018-31115-z)
Supplement: Supplementary file 1 — Supporting information [file 41598_2018_31115_MOESM1_ESM.docx]

Colloidal nitrogen is an important and highly-mobile form of nitrogen discharging into the Great Barrier Reef lagoon

Jonathan D. Judy,^1,2*^ Jason K. Kirby,^1^ Mark Farrell,^3^ Mike J. McLaughlin,^1^ Scott Wilkinson,^4^ Rebecca Bartley,^4^ and Paul M. Bertsch ^4^

**Contents:**

Four pages: S1-S3, 2 tables and 1 figure

***Page S3:***

**Table S1.** Soil type, parent geology and other characteristics of sampling areas.

***Page S3:***

**Table S2.** XRD analysis of WDC as a function of land use. Mineralogy reported as mean % composition ± one standard deviation (*n*=3). Gully data from analysis of samples from third cross-section from each gully.

**Figure S1**. Percent nitrogen in <0.45 µm fraction that was < 3kDa.

**Table S1.** Soil type, parent geology and other characteristics of sampling sites.

|  | **Grazed Gully sites 1-2** | **Grazed Gully Site 3** | **Reference Gully Sites 1-3** | **Sugarcane Sites 1-3** | **Sugarcane Site 4** |
| --- | --- | --- | --- | --- | --- |
| **Drainage basin** | Weany | Wheel | Main | Baratta Creek | Lower Burdekin |
| **Land use** | Grazing | Grazing | Non-agricultural | Sugarcane | Sugarcane |
| **Erosion feature sampled** | Gully wall and floor | Gully wall and floor | Gully wall and floor | Drainage feature near crop | Drainage feature near crop |
| **Catchment area (km^2^)** | 13 | 11 | 11 | 1,830 | 4,830 |
| **Mean rainfall**  **(mm yr^-1^)** | 690 | 740 | 780 | 900-1200 | 700-900 |
| **Median catchment gradient (%)** | 2.3 | 2.2 | 2.6 | <2% | <2% |
| **Soil type** | Chromosol | Chromosol | Chromosol | Sodosol and Dermosol | Chromosol |
| **Parent Geology** | Granite and Granodiorite | Granodiorite | Granite and Granodiorite | Alluvium | Alluvium |
| **Predominant ground vegetation** | Indian couch (*Bothriochloa pertusa*) | Indian couch (*Bothriochloa pertusa*) | Native perennial grasses, patches of exotic legumes and grasses | Sugarcane (*Saccharum officinarum)* | Sugarcane (*Saccharum officinarum)* |
| **Fertiliser application rate** | 0 | 0 | 0 | ~250 kg/ha/yr | ~250 kg/ha/yr |

**Table S2.** XRD analysis of WDC as a function of land use. Mineralogy reported as mean % composition ± one standard deviation (*n*=3). Gully data from analysis of samples from third cross-section from each gully.

|  | **SUGARCANE** | **UNDISTURBED** | | **GRAZED** | |
| --- | --- | --- | --- | --- | --- |
|  |  | **CHANNEL** | **WALL** | **CHANNEL** | **WALL** |
| **Mineral** | **MEAN ± SD** | **MEAN ± SD** | **MEAN ± SD** | **MEAN ± SD** | **MEAN ± SD** |
| **Quartz** | 4.5 ± 4.5 | <1 | <1 | <1 | <1 |
| **Smectite (Montmorillionite)** | 44.8 ± 14.8 | 38.7 ± 5.9 | 37.3 ± 24.2 | 42.0 ± 5.7 | 49.3 ± 10.5 |
| **Kaolinite** | 27.5 ± 5.4 | 46.3 ± 6.7 | 48.3 ± 17.5 | 43.3 ± 8.5 | 33.7 ± 11.2 |
| **Mica/Illite** | 11.5 ± 0.7 | 8.3 ± 2.3 | 9.3 ± 10.2 | 7.0 ± 2.1 | 10.7 ± 6.4 |
| **Plagioclase** | 2.8 ± 1.3 | <1 | <1 | 1 ± 0.0 | <1 |
| **Orthoclase** | 1.3 ± 0.5 | <1 | <1 | <1 | <1 |
| **Goethite** | 3.0 ± 1.4 | 6.7 ± 3.5 | 6.3 ± 3.1 | 5.0 ± 1.0 | 5.7 ± 3.2 |

**Figure S1**. Percent nitrogen in <0.45 µm that was < 3kDa. For gully samples, the three cross-sections were averaged to get a single data point for each gully. Error bars represent standard error
